# Role of CYLD in brain physiology and pathology

**DOI:** 10.1007/s00109-025-02521-4

**Published:** 2025-02-13

**Authors:** Leonardo Nardi, Frank Bicker, Jannik Maier, Ari Waisman, Michael J. Schmeisser

**Affiliations:** 1 Institute of Anatomy, University Medical Center of the Johannes Gutenberg-University, Mainz, Germany; 2 Focus Program Translational Neurosciences, University Medical Center of the Johannes Gutenberg-University, Mainz, Germany; 3 Institute for Molecular Medicine, University Medical Center of the Johannes Gutenberg-University, Mainz, Germany; 4 Research Center for Immunotherapy, University Medical Center of the Johannes Gutenberg-Universitys, Mainz, Germany

**Keywords:** Deubiquitinating enzyme, CYLD, Neurodegeneration, Inflammation

## Abstract

A common hallmark of several neuropsychiatric conditions is an altered protein homeostasis. In this context, ubiquitination has emerged as one of the most important post-translational modifications, regulating various intracellular processes such as protein degradation, autophagy, protein activation, and protein–protein interactions. Ubiquitination can be reversed by the activity of several deubiquitinating enzymes (DUBs), and it is of utmost importance that both processes remain in balance. Understanding the extent to which this system is involved in specific brain disorders opens up new possibilities for treating a broader spectrum of patients by targeting this central hub. In recent years, the attention to one of those DUBs, called CYLD, has increased sharply, but with relatively little focus on the central nervous system (CNS): 55 results for “CYLD Brain” vs. 895 results for “CYLD” in total (NCBI Pubmed search, 17.01.2025). Thus, we aim to provide a first overview of the new findings from the past decade specifically related to the role of CYLD in the physiology and pathology of the CNS.

## Introduction

*CYLD* was originally identified as a tumor suppressor gene mutated in familial cylindromatosis, a genetic condition characterized by the presence of multiple benign skin tumors that typically occur on the scalp and neck, termed cylindromas [1, 2]. The cells of those tumors are typically densely packed, forming characteristic cylinder-like structures, which are eponymous to the disease. It has also been reported that mutations in *CYLD* are implicated in multiple skin appendage tumors, including familial trichoepitheliomas and spiradenomas [3, 4], collectively unified under the umbrella term CYLD cutaneous syndrome [5].

**Fig. 1:**
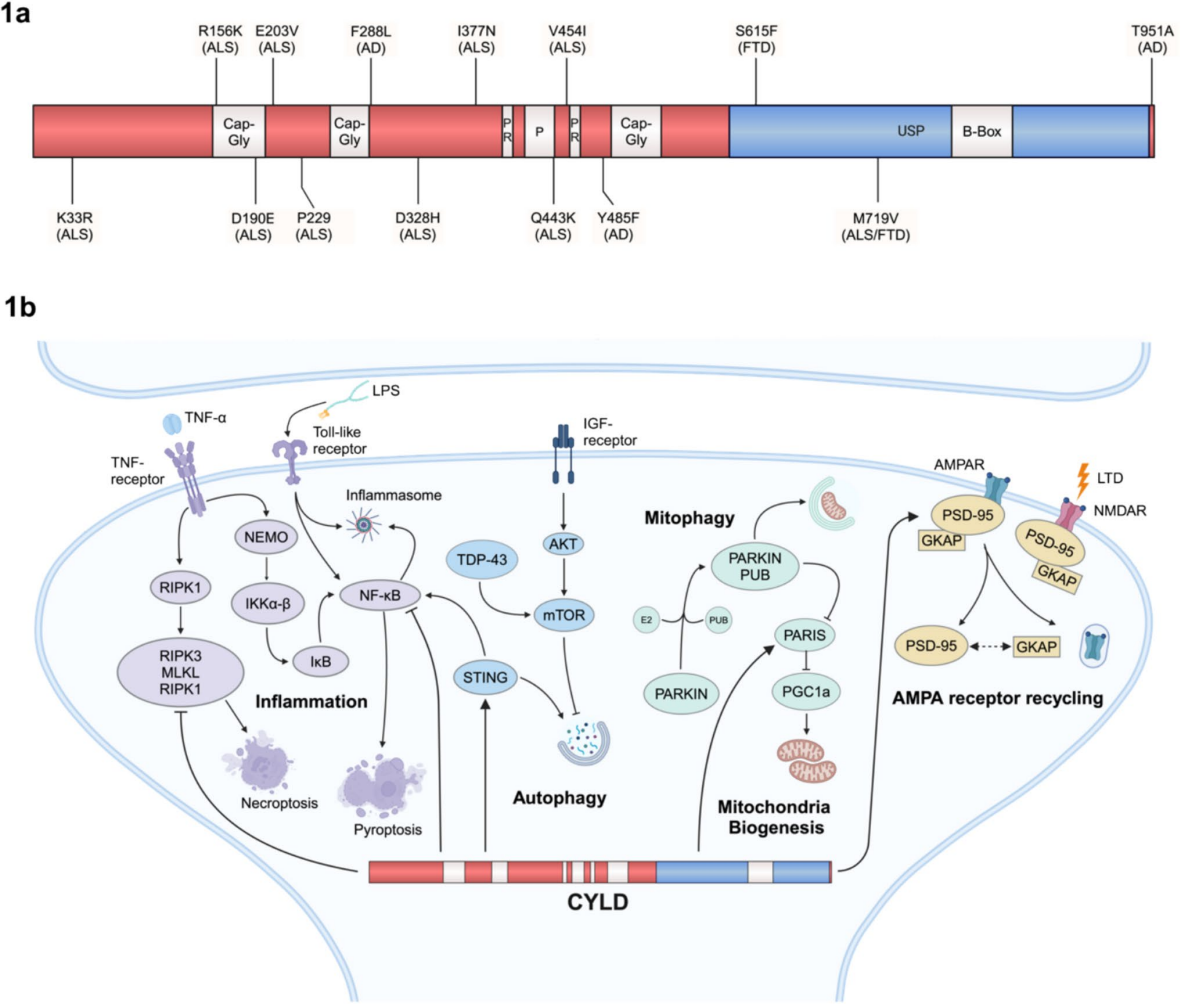
CYLD structure and involvement in neuronal-related cell signaling pathways. **a** Molecular structure of CYLD protein including functional domains and individual point mutations described in human patients (related disease represented in brackets). **b** CYLD directly affects a variety of signaling pathways within the postsynaptic specialization. Inflammation is regulated by CYLD via modulation of both NF-κb and TNF-α signaling (purple). CYLD is involved in autophagy by stimulating STING and inhibiting the AKT-mTOR pathway (blue). By regulating PARIS stability, CYLD participates to the interplay between mitophagy and mitochondrial biogenesis (green). Upon chemically induced LTD, CYLD deubiquitinates PSD-95 at K63, leading to its declustering and consequent AMPA receptor endocytosis (yellow, modified from [8])

*CYLD*, located on chromosome 16q12.1 (NCBI Gene ID: 1540), spans 56 kb and contains 23 exons, of which the first three are untranslated. Upon gene expression, 15 different splice variants are generated through posttranscriptional modifications, resulting in the translation of only three major protein isoforms. The full-length protein consists of 956 amino acids, has a molecular weight of approximately 120 kDa, and is highly conserved across a variety of species [6]. For example, the murine isoform shares 94.5% homology with the human isoform. Functionally, CYLD belongs to the subgroup of ubiquitin C-terminal hydrolases within the DUB family, which consists of more than 100 members in human. As such, the protein exhibits a ubiquitin-specific protease (USP) domain, enabling enzymatic interaction with polyubiquitinated proteins (Fig. 1a). Additionally, there is a zinc-finger-like B-Box domain within the USP domain, which is involved in subcellular localization. Beyond the USP domain, CYLD also includes three glycine-rich cytoskeleton-associated protein (CAP-Gly) domains responsible for microtubule binding [7], two proline-rich motifs required for protein–protein interactions, and a regulatory phosphorylation region [2].

Mechanistically, CYLD modifies lysine-63 (K63)-tagged effector proteins, thereby regulating protein–protein interactions, kinase function, and autophagy, rather than proteasomal degradation via K48-specific deubiquitination, thus affecting several intracellular pathways [9, 10] (Fig. 1b). In that way, CYLD acts as a negative regulator of the NF-κB signaling pathway, which is involved in inflammatory and immune responses, apoptosis, and tumorigenesis, by deubiquitinating TRAF2, TRAF6, and NEMO [11]. In turn, TNF-α-dependent activation of NF-κB induces CYLD, thereby creating an autoregulatory feedback loop. NF-κB exerts multiple roles in the CNS, including learning and memory. Interestingly, high activity of NF-κB has been detected in the hippocampus, amygdala, cerebellum, and cortex [12], all of which exhibit high levels of CYLD expression. In addition, members of the NF-κB pathway, such as CYLD, localize to the postsynaptic density (PSD) and are involved in the generation and maturation of neuronal spines [13] which will be discussed in more detail below. In the context of the CNS, CYLD’s role in the regulation of NF-κB could have various implications and may contribute to NF-κB’s action in neurodegenerative and neuroinflammatory diseases such as Alzheimer’s, Parkinson’s, and multiple sclerosis [14].

In addition to its regulatory function of NF-κB, CYLD is further involved in the regulation of WNT [15] signaling, a signal transduction pathway involved in various CNS functions, such as neurogenesis, tumorigenesis, and the progression of neurodegenerative diseases. Hence, it is not surprising that CYLD exhibits comprehensive biological relevance in the CNS, regulating a myriad of cellular functions including proliferation and cell death. However, it is important to note that the precise mechanisms and effects of CYLD in the brain are still under intensive research, and further studies are needed to achieve a comprehensive understanding of its roles in the CNS. For further details on the molecular mechanisms regulated by CYLD, we refer to the recent excellent review of Marin-Rubio et al. [6].

### CYLD in mouse brain

The first indication of a neural function for CYLD came in 2008, when it was shown that CYLD activity in neurons is regulated by p62/sequestome 1 (p62/SQSTM1), which modulates its interaction with TRAF6 [16]. However, it took another 5 years before Thein and colleagues demonstrated that CYLD accumulates at the PSD of neurons under physiological conditions [17]. Around the same time, initial publications began to explore CYLD’s contribution to the pathology of the CNS, particularly in the development of gliomas [18–20], which will be discussed further below. Since then, an increasing number of studies have investigated the functional role of CYLD in a neural context. However, expression of *Cyld* throughout the brain has been described only at the regional transcriptomic and proteomic levels (in both humans and mice), with high expression observed in the striatum, cortex, hippocampus, and thalamus [21–23]. Apart from rare exceptions, two *Cyld*^*−/−*^ mouse models have been predominantly used to investigate the impact of the DUB on brain structure and function [24, 25]. While research in this area is still in its early stages, phenotypical differences between the various *Cyld*^*−/−*^ models may reflect the different genetic targeting strategies employed.

The PSD, which serves as a central hub for communication between neurons in the CNS, faces the challenge of a constantly changing microenvironment. The complex task of receiving and processing vast amounts of information requires the accumulation and coordinated organization of specialized, heterogenous postsynaptic proteins. CYLD was initially identified as a component of the PSD in the CNS [26–29]. Both Western blotting and electron microscopy analyses revealed that CYLD accumulates significantly within PSD fractions of rat cerebral cortices and in rat primary hippocampal cultures following neuronal activity [27, 30]. Additionally, immunoblotting of hippocampal lysates and cultures indicated an accumulation of CYLD protein in the course of postnatal development [31]. In the same study, immunostainings further demonstrated a time-dependent enrichment of CYLD along developing dendrites. Overexpression of *Cyld* resulted in increased dendritic length and number of dendritic tips; CYLD knockdown resulted in reduced neurite length and complexity. Beyond neurite outgrowth, CYLD knockdown also resulted in reduced spine density, suggesting its role in spine morphogenesis. Similarly, another study showed that knockdown of CYLD in three different neuronal-like cells lines reduced neurite complexity. The same study also demonstrated a time-dependent increase in *Cyld* expression in the mouse cochlea, and interestingly, *Cyld*^*−/−*^ mice exhibited mild hearing loss [32].

The accumulation of CYLD upon depolarization, its localization along developing dendrites, and its impact on neurite and spine development underscore its importance for synaptic function. Notably, the activity-dependent recruitment of CYLD to the PSD has been traced to NMDA receptor activation [17], a process that depends on CYLD phosphorylation by CaMKII. Subsequent in vitro manipulations of PSD fractions linked CYLD phosphorylation at S-418 to the NF-κB signaling pathway [33]. Consistent with its expression pattern in the brain and its location at the PSD, Zhang et al. demonstrated impaired firing duration and rate in the striatum of *Cyld*^*−/−*^ mice [34]. Moreover, increased expression of the GABA_A_ receptor α1 subunit and the GABA_B_ receptor R2 subunit was observed in striatal lysates from *Cyld*^*−/−*^ mice, indicating that CYLD affects GABAergic activity. Since the striatum plays a key role in executive functions, these findings raised the question of whether the knockout of CYLD impacts behavior. While *Cyld*^*−/−*^ mice did not show significant differences from wildtype (WT) littermates in the open field test, increased anxiety-like behavior was observed in both the elevated plus maze and light–dark transition test [35]. Moreover, exposure of C57BL/6 J mice to chronic unpredictable stress for 21 consecutive days resulted in significantly reduced CYLD expression in the dorsolateral striatum, accompanied by impaired performance in anxiety-related behavioral tests, similar to the results seen in *Cyld*^*−/−*^ mice. A follow-up study demonstrated that *Cyld*^*−/−*^ mice displayed increased anxiety in the elevated plus maze following acute restrained stress. Interestingly, acute restrained stress resulted in divergent behavioral-responses between *Cyld*^*−/−*^ mice and their WT littermates: while WT mice spent less time in the open arms of the elevated plus maze, *Cyld*^*−/−*^ mice exhibited reduced anxiety-like behavior. Using c-fos staining, it was shown that after stress, the medial prefrontal cortex, dorsal striatum, nucleus accumbens, and basolateral amygdala exhibited the highest neuronal activation in these mice [36]. These results were supported by a further study showing impaired fear memory in *Cyld*^*−/−*^ mice [37, 38]. Fear-conditioning, paired with c-fos labeling, further highlighted the basolateral amygdala as a crucial region in an anxiety-dependent context. This finding is supported by electrophysiological alterations within the basolateral amygdala of *Cyld*^*−/−*^ mice, including reduced action potentials in response to current injection and altered excitatory and inhibitory synaptic activity. More detailed neuroanatomical investigations revealed a severe impact of CYLD loss on the morphology and firing rate of medium spiny neurons (MSNs), the most abundant cell type in the striatum [39]. Moreover, *Cyld* affects not only on the expression of GABA receptor subunits in the striatum and anxiety-related behavior but also the excitatory properties of striatal MSNs. Whole-cell patch-clamp experiments demonstrated a significant decrease in the amplitude and frequency of mini-EPSCs in MSNs, while measurements of AMPAR-mediated evoked EPSCs showed changes only in the amplitude of the measured signal. Furthermore, NMDAR-mediated evoked EPSCs remained unchanged between the genotypes. In line with these findings, CYLD deficiency led to a reduced amount of surface-expressed GluA1 and GluA2, while the NMDAR-related counterparts, NMDAR1 and NMDAR2B, remained unchanged between *Cyld*^*−/−*^ mice and controls.

Proteomic analysis of the striatum in a *Shank3* knockout and a *Shank3* transgenic mouse model showed that CYLD levels mirrored the gene dosage of *Shank3* [21]. Interestingly, altered expression of CYLD upon Shank3 gain- or loss-of-function was observed only in synaptosomal fractions. Following the generation of a CYLD interactome, it was concluded that SHANK3 regulates the synaptic localization of CYLD, rather than acting downstream as a CYLD substrate. Since SHANK3 is a key molecule related to various neuropsychiatric phenotypes (e.g., intellectual disability and repetitive behaviors), and shares a remarkable expression pattern with CYLD, the latter’s role in autism spectrum disorder (ASD) has been recently analyzed [40]. *Cyld*^*−/−*^ mice display aberrant social communication, cognitive impairment, and stereotypic, repetitive movements—all core phenotypes of ASD [41]. Further investigation of *Cyld*^*−/−*^ tissue revealed altered abundance of the autophagosome marker LC3B and total mTOR protein levels in hippocampal synaptosomes, linking CYLD to the process of autophagy. These findings are supported by a more recent study by Zajicek et al., which reported similar results in terms of LC3B protein abundance and mTOR hyperactivation [42].

In addition to major changes in striatal-related behavior upon CYLD deficiency, *Cyld*^*−/−*^ mice display alterations in hippocampal function. Similar to the striatal results, electrophysiological recordings of AMPA receptor–mediated mEPSCs in CA1 pyramidal neurons (PNs) revealed significant differences in amplitude between the genotypes [41]. This result was accompanied by impaired neuroanatomical morphology of PNs, including a reduced total dendritic length. Further studies emphasized the central role of CYLD in hippocampal excitability. Among other findings, disturbed fEPSPs were observed in the Schaffer collateral pathway of *Cyld*^*−/−*^ mice. Interestingly, alongside significant electrophysiological changes, a reduced fluorescence signal of the presynaptic protein vGlut1 was detected in the hippocampal stratum lacunosum moleculare [43]. However, a comparison of the outcomes in Colombo et al. and Chen et al. also revealed some deviations, which may be attributed to the different *Cyld*^*−/−*^ mouse lines used in each of these studies.

### Translational research about CYLD involvement in brain diseases

**Fig. 2:**
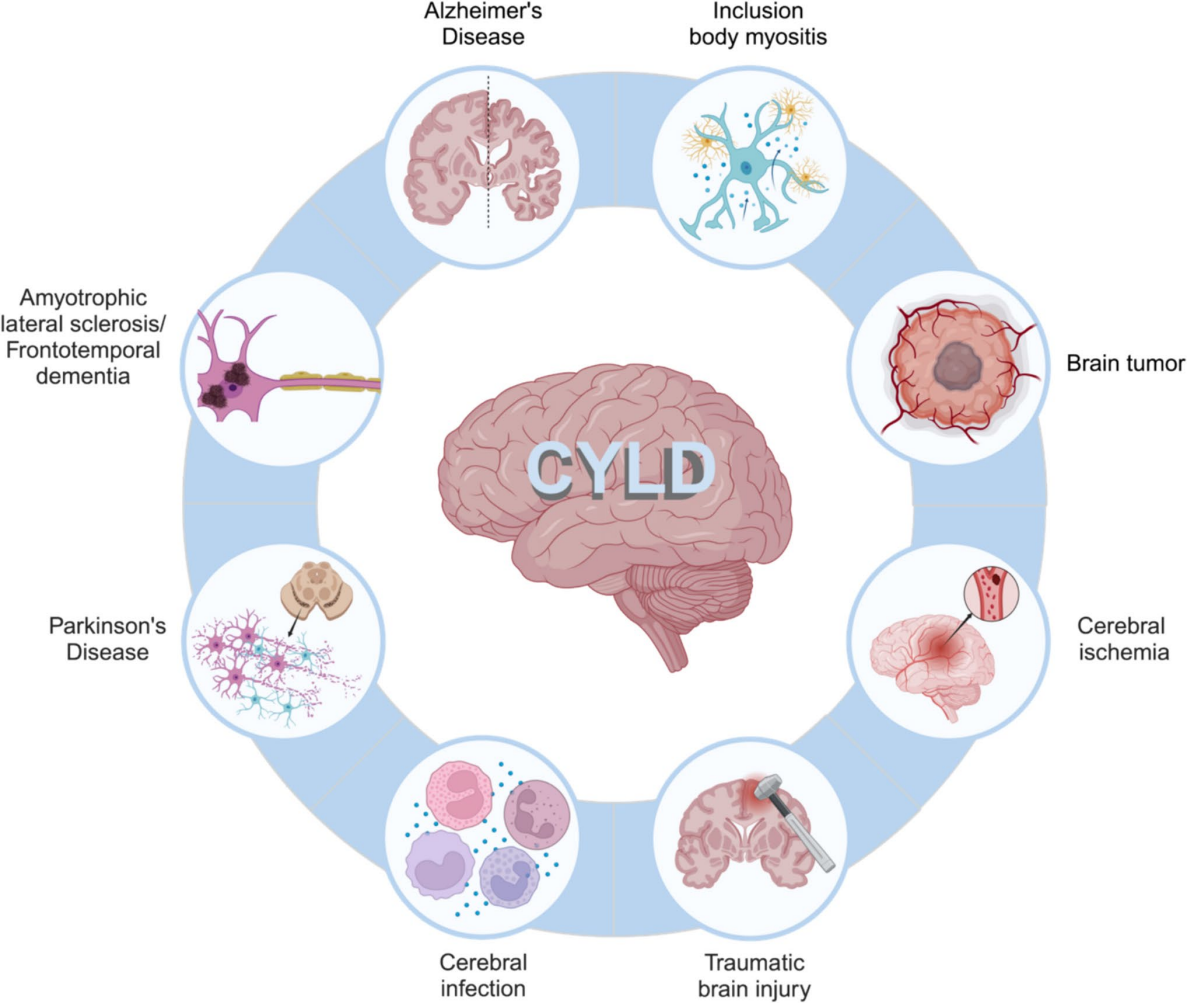
CYLD in brain diseases. Most abundant brain pathologies in which the available evidence shows an involvement of CYLD

Given the wide range of cellular pathways and functions regulated by CYLD, it is not surprising that it has been linked to several brain diseases. In this section, we will review the available evidence from both preclinical and clinical studies about the involvement of CYLD in the pathogenesis of neuropsychiatric conditions (Fig. 2).

#### Neurodegenerative disorders

A seminal study from 2020 reported a novel mutation in the catalytic domain of CYLD (Met719Val) in an Australian family affected by amyotrophic lateral sclerosis-frontotemporal dementia (ALS-FTD) [44]. Several cellular pathogenic effects of this mutation were characterized in vitro. In primary cortical neurons, overexpression of the Met719Val CYLD mutant resulted in a reduction in axonal length and branching. Moreover, it induced an increased localization of TDP-43 to the cytoplasm, a typical hallmark of some forms of ALS-FTD. Similar results were later reproduced in a non-neuronal cell line [45]. On an enzymatic level, the Met719Val CYLD mutant exhibited increased catalytic activity, as observed both in K63-directed deubiquitination and in the reduction of NF-κB activity. Although the interaction with key autophagy-related proteins (p62/SQSTM1, OPTN and TBK1) was not affected, the number of autophagosomes in cells transfected with the Met719Val CYLD mutant was increased, pointing at an impairment of autophagy. Interestingly, mutations in p62/SQSTM1, OPTN, and TBK1 are known to be associated with the pathogenesis of ALS-FTD and are characterized by autophagy impairments [46]. In the non-neuronal L929 cell line, expression of the Met719Val CYLD mutant led to increased cell death [47]. Shortly thereafter, two rare heterozygous variants (Ser615Phe and Pro229Ser) were reported in a Portuguese cohort of 65 FTD patients. Although the pathogenicity of these mutations was not demonstrated in vitro, the study highlighted a higher occurrence of CYLD mutations in FTD than previously reported [48]. In an additional Chinese cohort, consisting of 978 patients with sporadic ALS and 46 with familial ALS, 9 additional CYLD mutations were identified [49]. A more recent study even revealed novel CYLD mutations in Chinese patients affected by Alzheimer’s disease and FTD [50]. Another study highlighted the conserved role of CYLD across species in regulating dopaminergic neuronal survival in Parkinson’s disease. In Drosophila, knockdown of Parkin, PARIS, and PINK1, followed by CYLD knockdown, restored both motor deficits and dopaminergic neuronal survival. In case of PARIS overexpression, a phenomenon observed in some Parkinson’s disease cases [51], CYLD knockdown restored mitochondrial survival within dopaminergic cells. Similarly, the concomitant deletion of CYLD and Parkin increased the survival of mitochondria and dopaminergic neurons in both mouse and human embryonic stem cell models of Parkinson’s disease. Unlike ALS-FTD, autophagy was not affected in human embryonic stem cells deficient in Parkin alone or in both Parkin and CYLD, confirming the multifaceted effects of CYLD in the molecular pathomechanisms of different brain-related diseases [52].

#### Inclusion body myositis

Inclusion body myositis is a neurological condition characterized by both inflammatory and degenerative features. Although its pathogenesis is not fully understood, it shares common mechanisms with FTD, such as increased accumulation of cytoplasmic TDP-43 [53]. Both patients with inclusion body myositis and mice overexpressing TDP-43 exhibit increased co-localization of p-TDP43, p-p62, and K63-linked ubiquitin with CYLD in the muscle fibers [54].

#### Brain tumors

Given of CYLD’s tumor-suppressive role, it is not surprising to observe its involvement in the pathogenesis of brain tumors. Glioma subtypes with low CYLD expression are characterized by aggressive features, including reduced survival time, increased proliferation, vascularization, and invasion [20]. Moreover, glioma cells in hypoxic conditions downregulate CYLD expression [18]. Proteomic analysis of CYLD-knockdown glioma cells and RNA-seq data from glioblastoma samples revealed the involvement of WNT/β-catenin signaling in the development of the malignant phenotype associated to CYLD depletion. Inhibition of this pathway reversed the increased viability observed in CYLD-knockdown glioma cells [15].

#### Cerebral ischemia

As a negative regulator of the NF-κB pathway, CYLD plays a central role in inflammatory conditions such as neuronal death following stroke. Silencing CYLD results in increased survival of hippocampal cells (HT-22 cells) after cell death induction via TNF-α. Similar results were obtained by silencing RIP1, RIP3, or MLKL, which are components of the necroptosis signaling pathway [55]. In primary cortical neurons, CYLD expression increased progressively up to 12 h after oxygen–glucose deprivation, with caspase inhibitor ZVAD also being applied. Inhibition of the p38-SRF pathway using the p38 inhibitor SB203580 reduced both CYLD expression and cell death [56]. The studies presented so far suggest that silencing CYLD in vitro leads to a neuroprotective effect in the context of ischemia. In contrast, the following studies highlight a neuroprotective effect of CYLD in vivo. After middle cerebral artery occlusion/reperfusion, CYLD levels were elevated at 48 and 72 h in vivo. Overexpression of CYLD via lentivirus reduced the infarct area, decreased inflammatory markers (IL-1β and TNF-α), and improved behavioral outcomes after 72 h. In contrast, deletion of CYLD reduced the neuroprotective effects of electroacupuncture [57]. In a subsequent study, CYLD overexpression via lentivirus reduced pro-inflammatory markers and increased the anti-inflammatory microglial markers. It also dampened the expression of NLRP3 (inflammasome) in microglia cells. Interestingly, CYLD deletion via lentiviral delivery of shRNA led to the opposite changes in all the phenotypes observed. The anti-inflammatory effect of electroacupuncture was also diminished upon CYLD silencing [58]. In another stroke model (transient middle cerebral artery occlusion followed by reperfusion), SPATA2 was identified as a partner protein of CYLD, contributing to its recruitment. Deletion of SPATA2, followed by transient occlusion and reperfusion, led to reduced CYLD expression, increased activation of NF-κB signaling, and enhanced expression of NLRP3 (inflammasome) [59].

#### Traumatic brain injury

CYLD’s role in traumatic brain injury has also been revealed, particularly in the context of the necroptosis pathway. In vitro, silencing CYLD with siRNA improved cell survival after glutamatergic excitotoxicity. In vivo, *Cyld*^*−/−*^ mice exposed to traumatic brain injury showed reduced lesion volume at both 24 h and 7 days, along with reduced brain edema and intracranial pressure. Mechanistically, it was shown that CYLD regulates K63 deubiquitination, which in turn affects the availability of the RIP1/RIP3 complex, key components of the necroptosis signaling pathway [60].

#### Cerebral infection

In an experimental model of cerebral malaria, *Cyld*^*−/−*^ mice were protected compared to WT controls. The presence of CYLD led to increased proliferation of astrocytes and microglia, vascular damage with hemorrhage, and increased migration of CD8 + lymphocytes to the brain parenchyma. Depletion of CD8 + cells in WT mice resulted in a similar response to that observed in *Cyld*^*−/−*^ mice [61]. Interestingly, infection with HSV produced diametrically opposite effects in *Cyld*^*−/−*^ mice: survival was strongly reduced, and the viral titer was higher both in the spleen and the brain, suggesting that CYLD is essential for the viral response via the STING signaling pathway [62]. Astrocytes exposed to LPS showed increased markers of pyroptosis (NLRP3, ASC, cleaved caspase 1 and cleaved GSDMD) along with a time-dependent reduction in CYLD expression. *Cyld*^*−/−*^ mice, after LPS challenge, exhibited more astrocytes and greater expression of inflammation-related molecules (including inflammasome components and caspase 1) with reduced survival [63]. Selective overexpression of CYLD in microglia did not alter the response to LPS challenge [64].

#### Multiple sclerosis

Recent studies questioned the role of specific cell types in the pathogenesis of neuroinflammation related to multiple sclerosis. Although CYLD is known to regulate necroptosis in neurons [55, 60], it was not significantly altered in brain lysates from patients with MS compared to non-affected controls [65]. Moreover, both conditional overexpression of CYLD in microglia and general knockout did not affect the response to the induction of experimental autoimmune encephalitis, either at the cellular or behavioral level [64].

### Perspectives and impact

In this review, we have synthesized the growing body of evidence regarding the role of CYLD in CNS physiology and pathology. Although much remains unknown in this context, the increasing interest in DUBs, and CYLD in particular, reflects the growing attention of the neuroscientific community to this class of enzymes. By acting on a key posttranslational modification, the multifaceted CYLD can influence a wide variety of cellular processes, including autophagy, cell death induction, response to pathogens, and inflammation regulation. On the other hand, its pivotal role within the PSD underscores its significance in modulating neural function and synaptic plasticity, suggesting its potential relevance in the pathophysiology of neuropsychiatric disorders. The negative regulation of NF-κB signaling by CYLD has led to the hypothesis that this enzyme may be a key regulator of neuroinflammation. However, the evidence is mixed: both the abolition and overexpression of CYLD have resulted in pro- and anti-inflammatory effects in models of cerebral ischemia and infection. To better understand the role of CYLD in vivo, it will be necessary to use methods, such as the Cre-Lox approach, that allow for cell-type specific identification of the effects of selective deletion or enhancement.

Although evidence linking CYLD mutations to various neurodegenerative diseases is increasing, basic neurobiological research aimed at understanding the underlying mechanisms is still lacking, hindering the development of therapeutic approaches. Targeting CYLD might therefore represent a promising opportunity for developing novel therapeutic strategies to address the complex molecular mechanisms underlying neuropsychiatric disorders. In this regard, subquinocin is, to our knowledge, the first promising inhibitor of CYLD [66]. However, further research in the context of the CNS is still warranted.

## Data Availability

Not applicable.

## Abbreviations

ALS-FTD: Amyotrophic lateral sclerosis-frontotemporal dementia;
ASD: Autism spectrum disorder
CAP-Gly: Cytoskeleton-associated protein
CNS: Central nervous system
CYLD: Cylindromatosis
DUBs: Deubiquitinase(s)
MSNs: Medium spiny neurons
PNs: Pyramidal neuron
PSD: Postsynaptic density
USP: Ubiquitin-specific protease
WT: Wildtype
